# Design of the SILICOFCM study: Effect of sacubitril/valsartan vs lifestyle intervention on functional capacity in patients with hypertrophic cardiomyopathy

**DOI:** 10.1002/clc.23346

**Published:** 2020-03-03

**Authors:** Maria Tafelmeier, Andrea Baessler, Stefan Wagner, Bernhard Unsoeld, Andrej Preveden, Fausto Barlocco, Alessia Tomberli, Dejana Popovic, Paul Brennan, Guy A. MacGowan, Arsen Ristic, Lazar Velicki, Iacopo Olivotto, Djordje G. Jakovljevic, Lars S. Maier

**Affiliations:** ^1^ Department of Internal Medicine II (Cardiology, Pneumology, and Intensive Care) University Medical Centre Regensburg Regensburg Germany; ^2^ Medical Faculty, University of Novi Sad, Novi Sad Serbia and Institute of cardiovascular diseases of Vojvodina Sremska Kamenica Serbia; ^3^ Careggi University Hospital University of Florence Florence Italy; ^4^ Cardiology Department, Clinical Centre of Serbia, Faculties of Medicine and Pharmacy University of Belgrade Belgrade Serbia; ^5^ Cardiovascular Research, Clinical and Translational Research Institute Newcastle University and Newcastle upon Tyne Hospitals NHF Foundation Trust Newcastle upon Tyne UK

**Keywords:** familial cardiomyopathy, HCM, hereditary cardiac disease, left ventricular hypertrophy

## Abstract

**Background:**

Hypertrophic cardiomyopathy (HCM) is the most common genetic cardiovascular disease with a broad spectrum of disease severity. HCM ranges from a benign course to a progressive disorder characterized by angina, heart failure, malignant arrhythmia, syncope, or sudden cardiac death. So far, no medical treatment has reliably shown to halt or reverse progression of HCM or to alleviate its symptoms. While the angiotensin receptor neprilysin inhibitor sacubitril/valsartan has shown to reduce mortality and hospitalization in heart failure with reduced ejection fraction, data on its effect on HCM are sparse.

**Hypothesis:**

A 4‐month pharmacological (sacubitril/valsartan) or lifestyle intervention will significantly improve exercise tolerance (ie, peak oxygen consumption) in patients with nonobstructive HCM compared to the optimal standard therapy (control group).

**Methods:**

SILICOFCM is a prospective, multicenter, open‐label, randomized, controlled, three‐arm clinical trial (NCT03832660) that will recruit 240 adult patients with a confirmed diagnosis of nonobstructive HCM. Eligible patients are randomized to sacubitril/valsartan, lifestyle intervention (physical activity and dietary supplementation with inorganic nitrate), or optimal standard therapy alone (control group). The primary endpoint is the change in functional capacity (ie, peak oxygen consumption). Secondary endpoints include: (a) Change in cardiac structure and function as assessed by transthoracic echocardiography and cardiac magnetic resonance (MRI imaging), (b) change in biomarkers (ie, CK, CKMB, and NT‐proBNP), (c) physical activity, and (d) quality of life.

**Results:**

Until December 2019, a total of 41 patients were recruited into the ongoing SILICOFCM study and were allocated to the study groups and the control group. There was no significant difference in key baseline characteristics between the three groups.

**Conclusion:**

The SILICOFCM study will provide novel evidence about the effect of sacubitril/valsartan or lifestyle intervention on functional capacity, clinical phenotype, injury and stretch activation markers, physical activity, and quality of life in patients with nonobstructive HCM.

## 1 INTRODUCTION

Hypertrophic cardiomyopathy (HCM) is the most common inherited cardiovascular disease that affects approximately one in 500 of the general population.^1^, ^2^ Despite of advanced cardiac imaging, HCM is still under‐recognized in clinical practice and its initial diagnosis is often delayed.^2^ Approximately one third of patients with HCM have the nonobstructive form of the disease that was shown to be associated with a frequently underestimated adverse outcome.^1^, ^3^

The clinical diagnosis of HCM is based on left‐ventricular hypertrophy without cavity dilatation that cannot be explained by another cardiac, systemic, metabolic, or syndromic disease.^2^, ^4^, ^5^, ^6^ The course of HCM is highly variable, ranging from an asymptomatic, benign condition with a normal life expectancy to an advanced disease characterized by angina, dyspnea, heart failure, atrial fibrillation, malignant arrhythmia, syncope, or sudden cardiac death.^2^ Disease progression in nonobstructive HCM is associated with increasing myocardial fibrosis, microvascular ischemia, and abnormal cardiac function.^3^

The predominant cause are mutations of genes that encode protein components of the cardiac sarcomere and are transmitted in an autosomal‐dominant pattern.^1^ The mechanisms that lead from a sarcomere gene mutation to the phenotypic expression of HCM are poorly understood, which impedes the search for a treatment that can disrupt this pathophysiological process.^7^

So far, no medical treatment has reliably shown to prevent, halt, or reverse disease progression and targeted pharmacologic options are scarce.^8^ Clinical trials demonstrated limited or no effect of angiotensin receptor blockers or late sodium current inhibitor on disease progression, cardiac structure and function, exercise tolerance, and quality of life in patients with HCM.^9^ Accordingly, treatment recommendations are focused on the alleviation of symptoms, prevention of thromboembolic events, and the prophylactic implantation of cardioverter‐defibrillators in patients at high‐risk of sudden cardiac death.^4^, ^6^

The angiotensin receptor neprilysin inhibitor (ARNI) sacubitril/valsartan is a novel treatment shown to reduce hospitalizations and mortality in heart failure with reduced ejection fraction,^10^ while there was no significant benefit of sacubitril/valsartan on the rate of total hospitalizations for heart failure and cardiovascular death among patients with heart failure with preserved ejection fraction in the recently published PARAGON‐HF trial.^11^

However, sacubitril/valsartan was shown to be more effective for the management of hypertensive patients, compared with an angiotensin receptor blocker.^12^ Moreover, new preliminary data suggest that sacubitril/valsartan improves exercise tolerance and left ventricular wall motion, while reducing markers of left ventricular wall stress.^13^ As sacubitril/valsartan has not yet been evaluated in HCM, this is the first clinical trial to investigate its effects on cardiovascular performance in patients with HCM.

Lifestyle intervention is safe and can improve symptoms and signs in patients with heart failure. Physical activity intervention is associated with a significant increase in exercise tolerance, but appears to have limited effect on measures of cardiac morphology or function in patients with HCM.^14^ Dietary supplementation with inorganic nitrate (ie, concentrated nitrate‐rich beetroot juice) improves exercise capacity, vasodilatation and cardiac output reserves while reduces arterial wave reflections, which are linked to a left ventricular diastolic dysfunction and remodeling.^15^, ^16^, ^17^ Combined physical activity and dietary nitrate intervention has not previously been evaluated in HCM.

## 2 METHODS

### 2.1 Study design

SILICOFCM is a prospective, multicenter, open‐label, randomized, three‐arm, controlled phase II clinical trial that is designed to evaluate potential benefits of a 4‐month pharmacological intervention (sacubitril/valsartan) vs lifestyle intervention in patients with nonobstructive HCM. The primary hypothesis and secondary hypotheses are presented in detail in Table 1.

**Table 1:** Primary and secondary hypotheses

Primary hypothesis	Secondary hypotheses
A 4‐month pharmacological (sacubitril/valsartan) or lifestyle intervention will significantly improve exercise tolerance (ie, peak oxygen consumption) in patients with nonobstructive hypertrophic cardiomyopathy (HCM) compared to the optimal standard therapy (control group).	1. A 4‐month pharmacological or lifestyle intervention will significantly improve the clinical phenotype in transthoracic echocardiography and cardiac MRI imaging (ie, magnitude or distribution of cardiac hypertrophy, degree of left ventricular outflow obstruction, systolic and diastolic function, and left ventricular wall motion) compared to the optimal standard therapy.
	2. A 4‐month pharmacological or lifestyle intervention will significantly improve injury and stretch activation markers (ie, CK, CKMB, and NT‐proBNP) compared to the optimal standard therapy.
	3. A 4‐month pharmacological or lifestyle intervention will significantly improve quality of life (as determined by the SF12‐v2‐, Minnesota Living with Heart Failure‐, and Hospital Anxiety and Depression‐questionnaires) compared to the optimal standard therapy.

There are five participating institutions: Newcastle University Medical School and Newcastle upon Tyne Hospitals NHS Foundation Trust, (UK, coordinator), University Medical Centre Regensburg (DE), Institute for Cardiovascular Diseases Vojvodina (RS), University of Belgrade Clinical Centre (RS), and Careggi University Hospital Florence (IT). The Steering Committee of SILICOFCM is composed of the five principal investigators at each participating institution (please see also Table S3). The Steering Committee is responsible for protocol development, protocol and regulatory compliance, safety of participants at each site, analysis and review of the data, and preparation of written reports for publication. Special emphasis is put on close a collaboration between the five participating institutions, which is ensured by monthly teleconferences and biannual personal meetings.

The study is approved by each participating institution Research Ethics Committee / Institutional Review Board and will be conducted within the principles of Good Clinical Practice and in accordance with the Declaration of Helsinki. The study is registered with http://clinicaltrials.gov (http://clinicaltrials.gov‐identifier: NCT03832660) and received funding from the European Union's Horizon 2020 Research and Innovation Programme. Obtained data will be shared with the project partners for joint analysis.

Recruitment was started in May 2019 and enrolment is expected to be completed in May 2021. As duration of follow‐up for each patient will be 7 months, data analysis will be finished by November 2021 and a final report will be available at the beginning of 2022.

### 2.2 Subjects

Stable outpatients with a history of nonobstructive HCM or borderline left ventricular hypertrophy are eligible if they have a left ventricular wall thickness of ≥15 mm or ≥13 mm in a first degree relative of someone with HCM and meet the inclusion and exclusion criteria listed in Table 2. Patients are screened for eligibility and informed consent is obtained from all eligible patients who are willing to participate in the study.

**Table 2:** Inclusion and exclusion criteria

Inclusion criteria
1. Adults ≥18 years of age
2. Confirmed diagnosis of hypertrophic cardiomyopathy
3. Agreement to be a participant in the study protocol and willing/able to return for follow‐up
4. Able to provide written informed consent
5. In women of childbearing age: Willingness to use a highly effective contraceptive method (failure rate per year <1%)
Exclusion criteria
1. Less than 3 months postseptal reduction therapy (surgery or catheter‐based intervention)
2. Clinical cardiac decompensation in the previous 3 months, defined as New York Heart Association class IV congestive heart failure symptoms
3. Resting blood pressure >180/100 mmHg or systolic blood pressure <100 mmHg
4. Hypotensive response to exercise testing (≥20 mmHg decrease of systolic blood pressure from baseline blood pressure or an initial increase in systolic blood pressure followed by a decrease of systolic blood pressure ≥20 mmHg)
5. Concomitant use of angiotensin converting inhibitors or angiotensin receptor blockers; patients previously receiving angiotensin‐converting enzyme inhibitor or angiotensin receptor blocker therapy will require a 36‐hour washout period before initiation of Sacubitril/Valsartan
6. Resting left ventricular outflow tract gradient >50 mmHg
7. Left ventricular ejection fraction <50% by echocardiography
8. Implanted pacemaker or cardio‐defibrillator in the last 3 months or scheduled
9. History of hyperkalemia (serum potassium >5.2 mmol/L)
10. Renal insufficiency with a glomerular filtration rate <30 mL/min per 1.73 m^2^
11. Present or planned pregnancy
12. Life expectancy less than 12 months
13. Patients with severe adipositas (adipositas permagna, body mass index >40 kg/m^2^)
14. History of exercise induced syncope or sustained ventricular arrhythmias
15. Inability to exercise due to orthopedic or other noncardiovascular limitations
16. Use of other investigational drugs at the time of enrolment
17. Concomitant treatment with aliskiren‐containing drugs; discontinuation of treatment with aliskiren‐containing drugs is required before initiation of Sacubitril/Valsartan
18. History of angiotensin converting inhibitors‐ or angiotensin receptor blockers‐induced angioedema or history of hereditary or idiopathic angioedema
19. Evidence of hepatic disease as determined by any one of the following: AST or ALT values exceeding 2× ULN, severe hepatic insufficiency (classification Child Pugh C), biliary cirrhosis, cholestasis (current or anamnesic evidence), history of hepatic encephalopathy, history of esophageal varices, or history of portocaval shunt
20. Any surgical or medical condition that in the opinion of the investigator may place the patient at higher risk from his/her participation in the study or is likely to prevent the patient from complying with the requirements of the study or completing the study
21. History of noncompliance to medical regimens and patients who are considered potentially unreliable
22. History or evidence of drug or alcohol abuse within the past 12 months
23. History of malignancy of any organ system (other than localized basal or squamous cell carcinoma of the skin or localized prostate cancer), treated or untreated, within the past 2 years, regardless of whether there is evidence of local recurrence or metastases
24. Life‐threatening or uncontrolled dysrhythmia, including symptomatic or sustained ventricular tachycardia and AF or atrial flutter with a resting ventricular rate >110 beats per minute

### 2.3 Protocol

#### 2.3.1 Screening

After providing signed informed consent, participants undergo a screening visit to document age, weight/height, vital parameters, current symptoms, medical and family history, as well as prior, and concomitant medication. A physical examination, a 12‐lead‐electrocardiogram and a transthoracic echocardiography are performed. All participants undergo progressive cardiopulmonary exercise testing (CPET) using a cycle ergometer to evaluate their exercise tolerance, that is, peak oxygen consumption, at baseline. A venous blood sample is drawn to examine a comprehensive panel of serum and whole blood markers (eg, electrolytes, blood count, markers of renal function, muscle and liver enzymes, NT‐proBNP, lipid profile, HbA1c, TSH, and markers of inflammation). Habitual physical activity (daily number of steps) is evaluated using pedometers. Quality of life is assessed by the Minnesota Living with Heart Failure Questionnaire, the Hospital Anxiety and Depression Scale as well as the SF12‐v2. At the time of enrolment, all participants must be on optimal standard therapy for HCM, conforming to contemporary guidelines such as the European Society of Cardiology guidelines. All cardiac medications must remain unchanged for at least 2 weeks before screening and randomization.

#### 2.3.2 Randomization

Eligible participants with signed informed consent are randomly allocated to the study groups—receiving either treatment with sacubitril/valsartan or lifestyle intervention—or the control group (optimal standard therapy) using consecutively numbered sealed envelopes. Randomization will be stratified by patients giving or declining informed consent for the optional cardiac MRI‐imaging. If 10 patients are randomized within the cardiac MRI‐stratum, no further patients will be added to this stratum.

#### 2.3.3 Pharmacological intervention

Participants who are allocated to the pharmacological study group and have previously received an angiotensin‐converting enzyme inhibitor‐ or angiotensin receptor blocker‐therapy will require a 36‐hour washout period before initiation of sacubitril/valsartan to reduce the risk of angioedema.^18^ The treatment period begins with the initial dosing of sacubitril/valsartan, followed by up‐titration every 14 days to the target dose of 97/103 mg twice daily. The three doses of sacubitril/valsartan available throughout the study are 24/26 mg (dose level 1), 49/51 mg (dose level 2), and 97/103 mg (dose level 3), each taken by mouth twice daily.

Doses may be adjusted based on overall safety and tolerability. If necessary, dose adjustments or elimination of concomitant medications is made to alleviate adverse effects. If adverse effects are not alleviated or it is not possible to adjust concomitant medications, the study treatment may be down‐titrated by one dose level—or, at the lowest dose, temporarily withdrawn—for 1 to 2 weeks. Participants may then be reassessed and the study treatment further down‐titrated every 1 to 2 weeks until being deemed stable. Once stability is achieved, the participant is re‐challenged with up‐titration to the target dose. In case of discontinuation of the study medication, the participant is advised to return to the clinic for an end‐of‐study visit. Participants undergo treatment for 4 months.

After enrollment into the study, all participants are instructed to notify the study site about any change or withdrawal of medication. Furthermore, all medication, medical procedures and significant nondrug therapies (eg, blood transfusions and physical therapy) administered after the patient has been enrolled into the study are recorded.

#### 2.3.4 Lifestyle intervention

A 4‐month lifestyle intervention in patients with HCM consists of two integrated components, that is, physical activity and dietary supplementation with inorganic nitrate. In brief, the physical activity component aims to increase the patients' daily activity level by at least 2000 steps/day from baseline (eg, walking for approximately 30 minutes) at least 5‐7 days per week. To control for exercise intensity, patients will be instructed to use the standardized Borg Rating of Perceived Exertion Scale (6–20) to rate perceived exertion and to aim for levels between 11 and 14 (fairly‐light to somewhat hard; please refer to the online supplement and Table S1 for details). The exercise prescription will be progressed individually as conditioning takes place. No strength training or burst activity will be prescribed, all activities will fall well within the recommended national guidelines for recreational exercise^19^ and all participants are counseled to be alert to warning signs and symptoms that should prompt them to stop exercising and to seek medical advice.

The second component of the lifestyle intervention is a dietary supplementation with inorganic nitrate with a single dose of inorganic nitrate (NO_3−_) given in the form of concentrated nitrate‐rich beetroot juice (NO_3−_, BEET IT Sport, James White Drinks Ltd., Ipswich, United Kingdom) containing 6 mmol of NO_3−_ in a 70 mL bottle. Instructions will be provided for self‐administration of the nutritional intervention and patients will be asked to consume the beetroot juice each morning with the breakfast for 4 months. The EPIC‐Norfolk Food Frequency Questionnaire (please refer to the online supplement for details) will be administered at baseline and the Food frequency questionnaire EPIC Tool for Analysis (FETA) software used to extract dietary (energy and nutrient) information.^20^ Adherence to the lifestyle intervention will be tracked by completion of activity logs and self‐reported diaries, weekly telephone follow‐ups, as well as pedometers.

#### 2.3.5 Optimal standard therapy (control group)

Participants in the control group receive optimal standard therapy for HCM as advocated in the current guideline of the European Society of Cardiology.^6^ Optimal standard therapy may include an appropriate lifestyle, adequate management of heart failure symptoms and atrial fibrillation as well as risk stratification and preventive measures against sudden cardiac death.

#### 2.3.6 Echocardiogram

A transthoracic echocardiogram is performed by qualified cardiologists who are blinded to the participants' treatment allocation. Each study includes M‐mode and 2‐D images obtained from the standard parasternal and apical windows with three cardiac cycles being recorded. The echocardiogram—comprising color and tissue doppler—is done at rest and in response to the Valsalva maneuver, in order to detect a potential left ventricular outflow tract obstruction. Biplane Simpson's method is used for measurement of left ventricular (LV) end‐diastolic and end‐systolic volumes from which LVEF is derived. Details on the echocardiogram protocol and all assessed echocardiogram parameters are summarized in the online supplement and in Table S2.

#### 2.3.7 Cardiopulmonary exercise testing

A progressive CPET with a bicycle ergometer is performed to obtain a comprehensive assessment of pulmonary, cardiac, and circulatory function. At rest, during the exercise and throughout the recovery phase, an ECG is continuously monitored, and blood pressure and heart rate are frequently measured. At first, resting baseline values are recorded for 3 minutes. Then, the participants start pedaling for 3 minutes without any resistance at a pedal frequency of 60‐70 rpm, followed by a continuously increasing resistance at the predetermined ramp rate of 10 W/minute. The CPET is terminated, if complications (ie, significant ST‐segment deviation) arise, when the patient is unable to maintain a pedal‐frequency above 60 rpm or voluntary stops the exercise due to severe symptoms, such as breathlessness or exhaustion. Please refer to the online supplement for details.

#### 2.3.8 Electrocardiogram and ECG holter monitoring

The ECG will be performed using a standard 12‐lead‐electrocardiogram in supine position. To identify sporadic arrhythmia, all participants are asked to wear an ECG‐holter‐monitor for 24 hours and to keep a diary of activities and symptoms. The ECG‐holter‐recording is then correlated with the participants' activities and symptoms.

#### 2.3.9 Assessment of quality of life and habitual physical activity

Participants are asked to complete the validated and self‐administered SF12‐v2^21^‐ and Minnesota Living with Heart Failure‐questionnaires^22^ and the Hospital Anxiety and Depression Scale^23^ at baseline and as part of the clinic visits after 4 and 7 months, in order to assess general and disease specific quality of life. Permissions to use the SF12‐v2‐ and Minnesota Living with Heart Failure‐questionnaires and the Hospital Anxiety and Depression Scale were obtained from the copyright holders. Please refer to the online supplement for details on the assessment of general and disease‐specific quality of life. Habitual physical activity will be evaluated using pedometer for counting daily number of steps over a 7‐day period.

#### 2.3.10 Optional genetic testing

Participants who have given written informed consent are offered an optional genetic testing for mutations in genes associated with HCM. An investigator, who is well‐qualified in cardiac genetic counseling, clarifies what genetic testing might reveal and how family members might benefit from that knowledge. If participants choose to be genetically tested, they will be asked to provide written informed consent.

The genetic testing requires a blood sample and is performed at the Institutes of Human Genetics at the University Hospital Regensburg. The gene panel includes polymerase chain reaction (PCR)‐based assays to detect the following 34 genes, including all common and most rare variants with known clinical significance: ACTC1, ACTN2, ANKRD1, CALR3, CASQ2, CAV3, CRYAB, CSRP3, DES, FHL1, FLNC, GAA, GLA, JPH2, LAMP2, LDB3, MYBPC3, MYH6, MYH7, MYL2, MYL3, MYLK2, MYOZ2, MYPN, NEXN, PLN, PRKAG2, TCAP, TNNC1, TNNI3, TNNT2, TPM1, TTR, VCL. Genetic findings will be discussed with the patients in detail and—in case of positive results—patients will be invited for genetic counseling to identify relatives at an increased risk for HCM.

#### 2.3.11 Optional cardiac MRI‐imaging

Participants who have given written informed consent are offered an optional cardiac MRI‐examination. If patients choose to participate in the cardiac MRI‐examination, they will be asked to provide written informed consent. The cardiac MRI‐examination is implemented to evaluate cardiac morphology as well as systolic and diastolic function. Perfusion imaging is performed to assess first‐pass perfusion during bolus injection of 0.1 mmol/kg of gadolinium contrast agent. Phase sensitive 2D inversion‐recovery prepared gradient Echo imaging will be done 15‐20 minutes post injection to evaluate for delayed enhancement of the myocardium. Please refer to the online supplement for details on cardiac MRI imaging.

#### 2.3.12 Follow‐up

**Figure 1:**
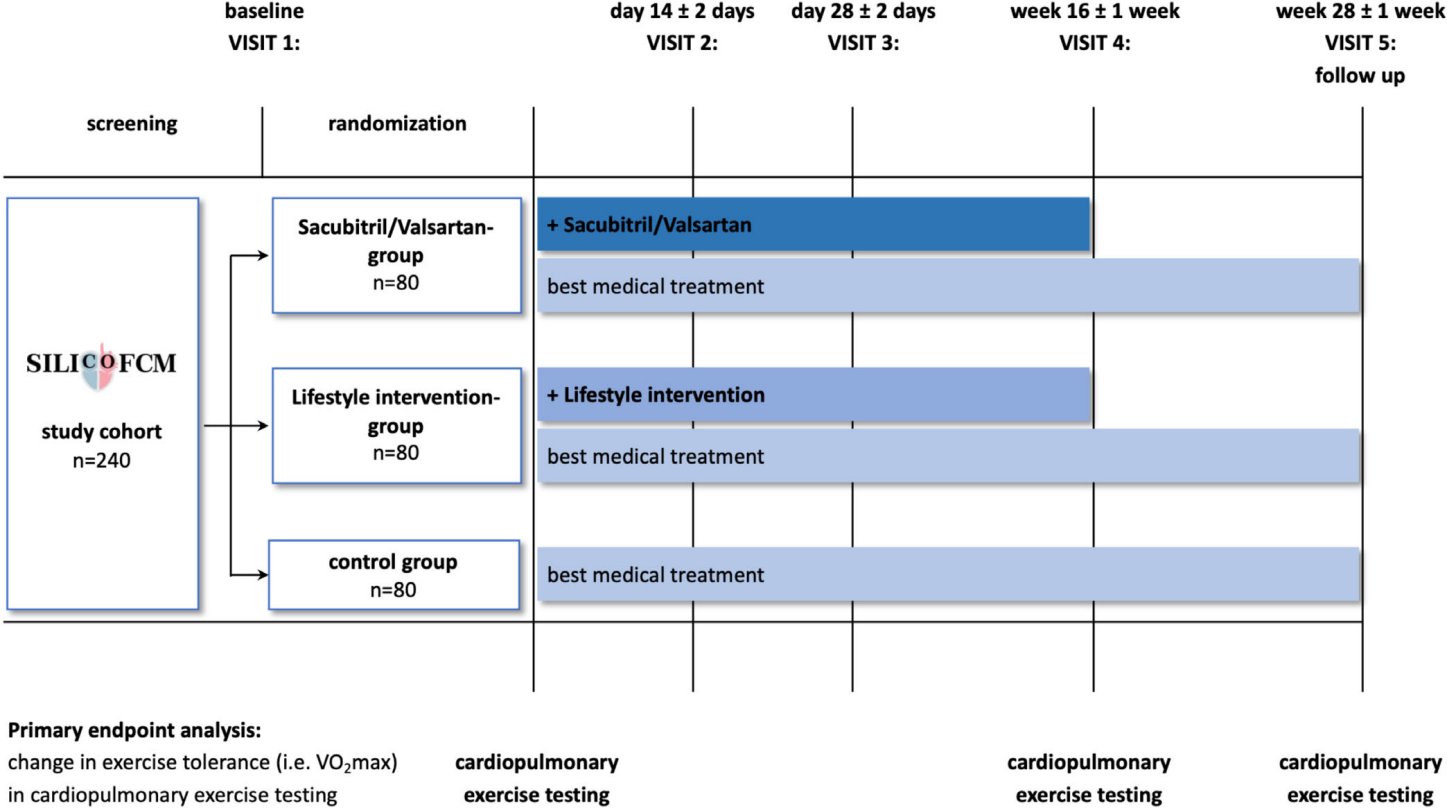
Study flow chart

The study schedule for participants is illustrated in Figure 1. Following screening and randomization, all participants attend clinic visits after 4 and 7 months, during which the assessments performed at baseline are replicated (Table 3). Moreover, participants in the study group are offered two additional visits to ensure medicinal safety before up‐titrating the sacubitril/valsartan‐dose after approximately 14 and 28 days. To detect the most common side effects of sacubitril/valsartan, that is, symptomatic hypotension, hyperkalemia and renal impairment, a physical examination is performed, current symptoms and concomitant medication are reviewed, and a blood sample is assessed for potassium and markers of renal function.

**Table 3:** Study schedule

Trial period	Screening/Randomization ± Start of Sacubitril/Valsartan/Lifestyle intervention	Intervention		Discontinuation of Sacubitril/Valsartan/Lifestyle Intervention	Follow up	Premature treatment discontinuation
Visit number/title	Visit 1	Visit 2	Visit 3	Visit 4	Visit 5	Discontinuation visit
Schedules days/week/month	day 1	Day 14	Day 28	Week 16 (month 4)	Week 28 (month 7)	At time of permanent treatment discontinuation
Scheduling Window Days/Weeks/Months:		± 2 days	± 2 days	± 2 weeks	± 2 weeks	
Administrative procedures						
Informed consent	x					
Informed consent for optional genetic testing (if applicable)	(x)					
Informed consent for optional Cardiac‐MRI imaging (if applicable)	(x)					
Inclusion criteria/Exclusion criteria	x					
Treatment randomization	x					
Clinical procedures/Assessments						
Weight/height	x			x	x	x
Vital signs (blood pressure, pulse etc.)	x	x	x	x	x	x
Physical exam	x	x	x	x	x	x
Symptoms	x	x	x	x	x	x
Medical history	x					
Family history	x					
Prior and concomitant medication review	x	x	x	x	x	x
Electrocardiogram (12‐lead)	x			x	x	x
Transthoracic echocardiogram	x			x	x	x
Cardiopulmonary exercise testing	x			x	x	x
ECG holter monitoring	x			x		
Blood work	x	x	x	x	x	x
Quality of life questionnaires	x			x	x	x
Adverse events monitoring	x			x	x	x
Optional genetic testing (if applicable)	(x)					
Optional Cardiac‐MRI imaging for imaging sub‐study (if applicable)	(x)			(x)		

Between planned visits, an extra clinic visit may be required when: (a) the patient reports an adverse event that in the investigator's opinion warrants further assessment, or (b) the patient irreversibly discontinues the study medication with sacubitril/valsartan, if tolerability issues cannot be alleviated.

#### 2.3.13 Outcomes

The primary study endpoint is the change in exercise tolerance (ie, peak oxygen consumption, peak VO_2_) in CPET. Secondary endpoints include: (a) Change in clinical phenotype in transthoracic echocardiography and cardiac MRI imaging (ie, magnitude or distribution of cardiac hypertrophy, degree of left ventricular outflow obstruction, systolic and diastolic function, and left ventricular wall motion), (b) change in injury and stretch activation markers (ie, Creatine kinase (CK), creatine kinase‐myocardial band (CKMB), and NT‐proBNP), (c) change in habitual physical activity, and (d) change in quality of life (as determined by the SF12‐v2‐, Minnesota Living with Heart Failure‐Questionnaire, and Hospital Anxiety and Depression Scale).

## 3 STATISTICAL ANALYSIS

### 3.1 Sample size and power calculation

The aim of the present study is to provide novel evidence about the efficacy and safety of sacubitril/valsartan vs lifestyle intervention in patients with nonobstructive HCM. In the absence of preliminary data regarding expectable treatment effects, we aim to enroll a total of 240 participants randomized into one of the three study arms. The average peak oxygen consumption in patient with HCM is suggested to be approximately 22 mL/kg/min.^14^ The same study in patients with HCM has demonstrated a 10% increase in peak oxygen consumption with 8‐12 weeks of lifestyle/exercise intervention.^14^ Assuming a similar effect, the present study plans to enroll 80 patients per intervention to have 90% power to detect a significant difference in peak oxygen consumption between the groups, at a two‐tailed significance level of 0.05 or less.

### 3.2 Analysis population

The primary analyses will be based on the intention‐to‐treat (ITT) analysis set, consisting of all patients who entered the study (ie, all patients who received a patient identification number). However, sensitivity analyses will be done on a per‐protocol (PP) analysis set that is composed of the ITT analysis set without major protocol violations. The latter serves to assess the robustness of the results. All safety data will be analyzed by means of the safety population of all study patients who had at least one post‐baseline safety assessment. The statement that a patient had no adverse events also constitutes a safety assessment.

### 3.3 Data analysis

Statistical analyses will be performed using SPSS software version 24.0 or higher (IBM SPSS Statistics, Armonk, New York). Data will be described as mean and SD for normally distributed data, as median (25.;75. percentile) for non‐normally distributed data and as numbers and percentages of participants for categorical variables. Differences in baseline characteristics, and outcome variables between the treatment‐ and control‐group will be compared using the analysis of variance (ANOVA) for normally distributed continuous variables, the Kruskal‐Wallis test for non‐normally distributed continuous variables and the Persons chi‐square test for categorical variables. A two‐sided *P*‐value of ≤.05 will be considered statistically significant for all analyses. As all secondary endpoints are of exploratory character, no adjustment for multiple testing will be performed.

## 4 RESULTS

Until December 2019, a total of 41 patients were recruited into the ongoing SILICOFCM study and were allocated to the study groups—receiving either treatment with sacubitril/valsartan (n = 14) or lifestyle intervention (n = 7)—and the control group (optimal standard therapy; n = 20). The mean age of the study population was 59.7 ± 13.4 years and 62% were male. Patients were diagnosed with hypertrophic nonobstructive cardiomyopathy with a mean maximum wall thickness of 19 ± 4 mm. There was no significant difference in key baseline characteristics between the three groups. Please refer to Table 4 for more details on key baseline characteristics.

**Table 4:** Key baseline characteristics

	Overall	Control group (optimal standard therapy)	Pharmacological intervention	Lifestyle intervention	*P*‐value
n (%)	41 (100)	20 (49)	14 (34)	7 (17)	
Age, years	59.7 ± 13.4	61.6 ± 13.9	60.3 ± 10.4	52.7 ± 16.5	0.315 ^A^
Male sex, n (%)	26 (62)	11 (55)	9 (64)	6 (86)	0.347 ^Chi^
Body mass index, kg/m^2^	27.7 ± 3.9	27.9 ± 4.4	26.5 ± 3.4	29.4 ± 3.4	0.286 ^A^
Left ventricular ejection fraction, %	64.1 ± 7.1	65.4 ± 6.6	63.9 ± 8.5	61.1 ± 5.3	0.410 ^A^
Maximal wall thickness, mm	19 ± 4	18 ± 4	21 ± 5	17 ± 2	0.092 ^A^
Resting LVOT gradient, mmHg	17.5 ± 15.3	17.1 ± 11.9	18.0 ± 23.5	18.0 ± 4.2	0.984 ^A^

*Note*: Key baseline characteristics of the study population of patients that were enrolled into the ongoing SILICOFCM study until December 2019 (n = 41) according to their allocation to the study groups—receiving either treatment with sacubitril/valsartan or lifestyle intervention—or the control group (optimal standard therapy). Data are presented as mean ± SD unless otherwise stated. Chi, Chi‐square test; A, ANOVA; LVOT, left ventricular outflow tract.

## 5 DISCUSSION

Until recently, nonobstructive HCM was predominantly regarded as a pathophysiologically benign disorder without significant symptoms, high‐risk characteristics or the necessity for major treatment options.^5^ However, nonobstructive HCM has been linked to increased morbidity due to a high risk for malignant ventricular arrhythmia^3^ that may be caused by myocardial fibrosis and microvascular ischemia.^24^ As treatment options in patients with nonobstructive HCM are usually restricted to pharmacological therapy, promising new drugs, such as sacubitril/valsartan, are essential and need to be investigated in clinical studies.

Lifestyle intervention has been shown to improve exercise tolerance in HCM but at the same time limited effect on cardiac structure and function was reported.^14^ Recent studies have demonstrated that dietary supplementation with inorganic nitrate improves exercise capacity and hemodynamic function in patients with heart failure preserved ejection fraction.^15^, ^16^, ^17^ The potential physiological effect of a combined physical activity and dietary nitrate on HCM phenotype has not been previously investigated and is subject to evaluation in the present SILICOFCM study.

Therefore, our randomized controlled clinical SILICOFCM trial will provide a novel evidence about the safety and efficacy of sacubitril/valsartan vs lifestyle intervention in patients with HCM. Secondly, several exploratory endpoints will complement primary analyses with regard to potential changes in clinical phenotypic characteristics, injury and stretch activation markers, and quality of life. Thirdly, comprehensive genetic analyses may further elucidate the complex genotype‐phenotype relationship characteristic of HCM and its impact on treatment response.

### 5.1 Assessment of sacubitril/valsartan in patients with nonobstructive HCM

Having once been considered as contraindicated due to concerns for potential hemodynamic complications, CPET is nowadays a well‐established clinical tool in the comprehensive evaluation of HCM patients.^25^, ^26^ The medical benefits of CPET in HCM comprise the differentiation of mild left ventricular hypertrophy from physiological hypertrophy,^27^ a better prediction of disease progression as well as the objective evaluation of therapeutic strategies and treatment effects.^25^, ^26^

The change in peak VO_2_ in CPET was chosen as primary endpoint of this study, because peak VO_2_ is considered a factual parameter for assessing exercise capacity in patients with HCM.^28^ Moreover, low peak VO_2_ is generally acknowledged as an independent predictor for major adverse events in HCM, while normal peak VO_2_ is associated with a favorable clinical outcome.^25^

Besides, the SILICOFCM trial covers a wide range of secondary outcome parameters, for example, clinical phenotypic characteristics, injury and stretch activation markers, quality of life, and physical activity, that will be assessed in an exploratory manner. Accordingly, the SILICOFCM aims to evaluate the effect of sacubitril/valsartan vs lifestyle intervention on nonobstructive HCM in a comprehensive way.

### 5.2 Potential mechanisms of action of sacubitril/valsartan intervention in nonobstructive HCM

The underlying pathophysiology and mechanisms of HCM lead to increased myocardial fibrosis and hypertrophy. This may be mediated by microvascular ischemia and an up‐regulated feedback loop of the renin‐angiotensin‐aldosterone system.^8^ By simultaneously blocking the renin‐angiotensin‐aldosterone system and augmenting natriuretic peptides through neprilysin inhibition,^29^ the ARNI sacubitril/valsartan promotes vasodilatation, natriuresis and inhibition of fibrosis and hypertrophy.^30^, ^31^ Thus, sacubitril/valsartan has already emerges as an innovative and promising therapeutic approach in heart failure with reduced ejection fraction.^10^

So far, the potentially beneficial effects of sacubitril/valsartan in HCM has not been evaluated in clinical trials. Therefore, first mechanistic insight must be derived from experimental data and a single case report: Sacubitril/valsartan was shown to attenuate left ventricular remodeling and dysfunction by inhibiting cardiac fibrosis and hypertrophy in both in vivo‐ and in vitro‐analyses.^29^ The first positive response to treatment with sacubitril/valsartan in HCM has been described in a case report by Rubis et al.^32^: a middle‐aged woman with nonobstructive end‐stage HCM was treated with sacubitril/valsartan for 3 months, which resulted in marked improvements in symptoms and exercise tolerance as well as an enhanced left ventricular function and decreased NT‐proBNP levels.^32^

### 5.3 Potential mechanisms of action of lifestyle intervention in nonobstructive HCM

While aerobic exercise training was shown to improve cardiovascular health and overall survival in the general population,^33^, ^34^ data on the safety and benefit of recreational exercise for patients with HCM is still scarce.^35^, ^36^ However, a study by Saberi et al. showed that moderate‐intensity exercise training over a period of 16 weeks in patients with HCM significantly improved their exercise capacity compared to usual activity.^14^ Most importantly, exercise training in patients with HCM was safe without any occurrences of sustained ventricular arrhythmia, sudden cardiac arrest, appropriate defibrillator shock or death.^14^

Recently, several reports demonstrated that dietary supplementation with NO_3−_—that is particularly abundant in beetroot juice—increases exercise capacity in patients suffering from heart failure with preserved ejection fraction.^15^, ^16^, ^17^ Ingested NO_3−_ is converted to nitrite (NO_2−_) by anaerobic bacteria in the oral cavity^37^ that is subsequently reduced to nitric oxide (NO) by metalloproteins (eg, deoxyhemoglobin or deoxymyoglobin).^38^ This conversion and delivery of NO occurs preferentially in the setting of hypoxemia and thus, may ultimately decrease systemic vascular resistance and increase cardiac output during physical exercise.^17^

### 5.4 Genetic testing

As a particular feature of the present study, all participants are offered comprehensive genetic testing for mutations in genes associated with HCM. Due to variable penetrance and expression,^39^ the phenotypic characteristics of HCM are multifaceted and may be influenced by other factors beyond single pathogenic mutations.^40^ The complex genotype‐phenotype characteristics of HCM were potentially under‐recognized in the past, which may have resulted in an unsatisfactory efficacy of drug management.^40^ Therefore, our clinical trial may contribute to a better understanding of the association between sarcomere mutations and phenotype characteristics and disease severity as well as its impact on treatment response.

## 6 LIMITATIONS

The design of the SILICOFCM has a few limitations to be considered, in particular the short treatment period. Preliminary data about expectable treatment effects of sacubitril/valsartan in patients with nonobstructive HCM is missing to date. The sample size of this trial was considered sufficient to get robust effect estimates in regard to the efficacy and safety of the study treatments. Participants undergo study treatment for 4 months, which was deemed sufficient to observe potentially beneficial effects of the complementary addition of sacubitril/valsartan to the optimal standard therapy of HCM. Comparable treatment durations with sacubitril/valsartan were previously shown to significantly reduce NT‐proBNP in patients with heart failure with preserved ejection fraction^41^ and to improve cardiac function in the above mentioned case report of a patient with HCM.^32^

Due to the above‐mentioned limitations, the results of our trial may have to be interpreted with caution. However, they may pave the way for future clinical trials on patients with nonobstructive HCM.

## 7 CONCLUSION

Over the past decade, we have gained a better understanding of the clinical management of patients with HCM. There have been considerable advances in treatment strategies directed at symptomatic relief and the prevention of sudden cardiac death. Henceforth, new approaches need to be evaluated regarding their potential to halt the phenotypic expression and progression of HCM.

In this aspect, SILICOFCM will provide novel data on whether the complementary addition of sacubitril/valsartan or lifestyle intervention to the optimal standard therapy improves cardiovascular performance in patients with nonobstructive HCM as well as their clinical phenotypic characteristics, injury and stretch activation markers, habitual physical activity, and quality of life. The SILICOFCM study has the potential to generate rational hypotheses regarding the effect of sacubitril/valsartan or lifestyle intervention on functional capacity in patients with nonobstructive HCM that then need to be confirmed in larger randomized clinical trials. Thus, SILICOFCM will clearly contribute to optimizing the management of patients with nonobstructive HCM and to improving their clinical outcome.

## CONFLICT OF INTEREST

This trial has received funding from the European Union's Horizon 2020 Research and Innovation Programme under Grant Agreement no. 777204.

## Supporting information


**Appendix S1.** Supporting information.
