# Supplementary material for: Design of the SILICOFCM study: Effect of sacubitril/valsartan vs lifestyle intervention on functional capacity in patients with hypertrophic cardiomyopathy
Source: Clin Cardiol. 2020 Mar 3;43(5):430–40. doi: 10.1002/clc.23346 (PMC7244301; doi:10.1002/clc.23346)
Supplement: Supplementary file 1 — Appendix S1. Supporting information. [file CLC-43-430-s001.docx]

**Design of the SILICOFCM study:**

**Effect of sacubitril/valsartan vs. lifestyle intervention on functional capacity in patients with hypertrophic cardiomyopathy - Online data supplement**

**Authors**:

Maria Tafelmeier^1^, Andrea Baessler^1^, Stefan Wagner^1^, Bernhard Unsoeld^1^, Andrej Preveden^2^, Fausto Barlocco^3^, Alessia Tomberli^3^, Dejana Popovic^4^, Paul Brennan^5^, Guy A. MacGowan^5^, Arsen Ristic^4^, Lazar Velicki^2^, Iacopo Olivotto^3^, Djordje G. Jakovljevic^5*^, Lars S. Maier^1*^

^*^ Both authors contributed equally to this manuscript.

**Assessment methods and questionnaires**

Borg Rating of Perceived Exertion Scale

The Borg Rating of Perceived Exertion Scale was developed by Gunnar Borg and is a user-friendly tool for measuring an individual’s self-rated physical exertion, breathlessness and fatigue during exercises with scores ranging from 6 (no exertion) to 20 (maximum exertion).^1^ The scale ranges from 6 to 20 as a simple way to estimate the heart rate, as multiplying the Borg-score by 10 gives an approximate heart rate for a particular level of activity.^1^ Patients are instructed to aim for moderate levels of exertion between 11 - 14 (fairly-light to somewhat hard) while increasing their daily activity level during lifestyle intervention. Please refer to e-Table 1 for details.

EPIC-Norfolk Food Frequency Questionnaire

The EPIC-Norfolk Food Frequency Questionnaire (EPIC-FFQ) is a self-completed semiquantitative food frequency questionnaire to assess an individual’s habitual diet during the past year.^2^ The EPIC-FFQ consists of two parts: The main part contains a list of 130 foods and for each item on the list, participants are asked to indicate their usual rate of consumption choosing from nine frequency categories. The second part focuses on further nutrition details, such as breakfast cereals, type of fat used in frying, roasting, grilling or baking or type and quantity of milk consumed.^2^

Assessment of general and disease-specific quality of life

- SF12-v2:

The SF12-v2 questionnaire is a valid and widely used instrument for assessing health-related quality of life.^3^ Being the abridged and more practical version of the 36-item Short Form Health Survey (SF-36)^4^, the SF12-v2-questionnaire comprises 12 items and covers the same eight health domains as the SF-36, namely physical functioning, role limitations due to physical problems, bodily pain, general health perception, vitality, social functioning, role limitations due to emotional problems and mental health.^3^

- Minnesota Living with Heart Failure questionnaire:

The self-administered Minnesota Living with Heart Failure Questionnaire (MLHFQ) is the leading method for assessing disease-specific quality of life questionnaires for patients with heart failure.^5,6^ The MLHFQ comprises 21 items that represent different degrees of impact of heart failure on disease-specific quality of life, from 0 (none) to 5 (very much). It provides a total score (range 0 - 105, from best to worst disease-specific quality of life), as well as scores for two dimensions, physical (8 items, range 0 - 40) and emotional (5 items, range 0 - 25).^6^

- The Hospital Anxiety and Depression Scale:

The Hospital Anxiety and Depression Scale (HADS) is a brief self-reporting two-dimensional questionnaire that was developed to screen for levels of anxiety and depression among patients in a general hospital setting.^7^ It can be divided into an Anxiety subscale (HADS-A) and a Depression subscale (HADS-D) both containing seven intermingled items that can be scored from 0 - 3.^7^

**Protocols**

Echocardiogram protocol

A transthoracic echocardiogram - comprising 2D-, M-mode-, color- and tissue-Doppler-imaging - is performed by qualified cardiologists who are blinded to the participants’ treatment allocation according to the guidelines of American Society of Echocardiography.^8^ In brief, real-time 2D-images are acquired in the standard parasternal (long-axis and short-axis views) and apical (apical 4-chamber, apical 2-chamber and apical long axis view) views, with three cardiac cycles being recorded. Parasternal long axis view of the right ventricular and right ventricular outflow tract is monitored. Parasternal short-axis views are acquired at 3 levels: basal (at mitral valve level), midpapillary, and apical (minimum cavity distal to papillary muscle level). Apical 4-chamber view recorded for left and right ventricular evaluation. Biplane Simpson’s method is used for measurement of left ventricular end-diastolic and end-systolic volumes from which left ventricular ejection fraction is derived. Peak velocity of the left ventricular outflow tract is recorded from the apical long axis view by pulse Doppler at rest and in response to the Valsalva maneuver, in order to detect a potential left ventricular outflow tract obstruction. Please refer to e-Table 2 for further details on all echocardiogram parameters that are collected in SILICOFCM.

Cardiopulmonary exercise testing protocol

A cardiopulmonary exercise testing (CPET) using a bicycle ergometer is performed to obtain a comprehensive assessment of oxygen consumption and ventilation, cardiac, and circulatory function. Patients are asked to wear a facemask to collect and analyze ventilation parameters and expired respiratory gases. Moreover, cardiac output is measured using a non-invasive bioreactance method that utilizes four dual electrodes placed on the back side of the patient’s thorax. At rest, during the exercise and throughout the recovery phase, an ECG is continuously monitored, and blood pressure and heart rate are frequently measured.

At first, resting baseline values are recorded for 3 minutes. Then, the patients start pedaling for 3 minutes without any resistance at a pedal frequency of 60-70 revolutions per minute (rpm), followed by a continuously increasing resistance at the predetermined ramp rate of 10 Watt/minute. Respiratory gas exchange data are collected continuously, and the Borg Rating of Perceived Exertion Scale is performed every 3 minutes. The CPET is terminated, if complications (i.e. significant ST-segment deviation) arise, when the patient is unable to maintain a pedal-frequency above 60 rpm or voluntary stops the exercise due to severe symptoms, such as breathlessness or exhaustion (as indicated by respiratory exchange ratio > 1.1, Borg Rating of Perceived Exertion Scale > 18 or absence of rise in oxygen consumption with further increase in exercise intensity). The anaerobic threshold is determined using the V-slope method.

Cardiac MRI-imaging protocol

Using a 3-Tesla Intera Achieva scanner (Philips, Best, NL), subjects undergo (i) cine imaging, (ii) cardiac tagging, and (iii) late gadolinium enhancement (LGE) imaging.

Using a 3-Tesla MRI-scanner, patients undergo cardiac MRI-imaging with (i) cine imaging, (ii) cardiac tagging, and (iii) late gadolinium enhancement (LGE) imaging to evaluate cardiac morphology as well as systolic and diastolic function.

- Cine imaging:

Patients are scanned supine using a 6-channel cardiac coil and ECG gating. The short-axis balanced steady-state free precession images are obtained covering the entire left ventricle [field of view (FOV) 350 × 350 mm^2^, repetition time/echo time (TR/TE) = 3.7/1.9 ms, turbo factor 17, flip angle (FA) 40^o^, slice thickness 8 mm, 25 phases, resolution 1.37 mm]; long-axis images are acquired. Endocardial and epicardial borders are traced manually on short-axis slices throughout the cardiac cycle. The LV mass, and systolic and diastolic parameters, including the ratio of early to late ventricular filling velocity (E/A ratio) and early filling percentage, are calculated. The ratio of the LV mass to end-diastolic volume (M/V ratio) is calculated as an index of concentric hypertrophy. Longitudinal shortening is determined in the four-chamber view as the percentage difference in distance from the mitral valve plane to the apex at end-systole and end-diastole. The myocardial wall thickness is determined at the same level as tagging, and the percentage increase from diastole to systole (radial thickening) is calculated.

- Cardiac tagging:

MR signal from the myocardium in diastole is cancelled in a rectangular grid pattern and tags are tracked through the cardiac cycle. A multi-shot turbo-field echo sequence is used (TR/TE/FA/number of averages = 4.9/3.1/10^o^/1, turbo factor 9, SENSE factor 2, FOV 350 × 350 mm^2^, voxel size 1.37 mm, tag spacing 7 mm, 12 phases). Two adjacent short-axis slices of 10 mm thickness are acquired at the mid-ventricle with a 2-mm gap. The Cardiac Image Modeling package (Auckland UniServices Ltd, Auckland, New Zealand) is used to align a mesh on the tags between the endocardial and epicardial contours. Peak circumferential strain for both the whole myocardial wall and the endocardial third are calculated. Peak torsion between the two slices is calculated as the circumferential-longitudinal shear angle, defined on the epicardial surface.

- LGE imaging:

Gadolinium-DTPA of 0.2 mmol/kg is administered intravenously to patients. LGE images are obtained at 10 min using a breath-held, cardiac-triggered three-dimensional phase-sensitive inversion recovery sequence (multi-shot gradient echo TR/TE = 5/2.4, FA = 15^o^/5^o^, acceleration factor 31, parallel imaging factor 2, 1.8-mm resolution zero-filled to 1.3 mm) with the inversion time to null normal myocardium determined from a prior multi-slice 2D Look-Locker experiment (multi-shot EPI with an EPI factor 5, acceleration factor 2, TR/TE = 7.3/2.8, 3-mm resolution). Qualitative analysis for the presence and distribution of LGE in a 17-segment model was performed by two experienced, independent observers, blinded to patient status and other findings: consensus agreement is planned in the case of initial disagreement between observers.

**e-Tables**

**e-Table 1** The Borg Rating of Perceived Exertion Scale

| **How you might describe your exertion:** | **The Borg Rating of Perceived Exertion Scale** | **Examples** |
| --- | --- | --- |
| None | 6 | Reading a book, watching television |
| Very, very light | 7 to 8 | Tying shoes |
| Very light | 9 to 10 | Chores like folding clothes that seem to take little effort |
| Fairly light | 11 to 12 | Walking through the grocery store or other activities that require some effort but not enough to speed up your breathing |
| Somewhat hard | 13 to 14 | Brisk walking or other activities that require moderate effort and speed your heart rate and breathing but don’t make you out of breath |
| Hard | 15 to 16 | Bicycling, swimming, or other activities that take vigorous effort and get the heart pounding and make breathing very fast |
| Very hard | 17 to 18 | The highest level of activity you can sustain |
| Very, very hard | 19 to 20 | A finishing kick in a race or other burst of activity that you can’t maintain for long |

**e-Table 2** Echocardiogram data sheet

| **date of echocardiography** | _ _. _ _. _ _ _ _ | |  |
| --- | --- | --- | --- |
|  |  |  |  |
| **study identification** |  |  |  |
|  |  |  |  |
| **weigh** |  | kg |  |
| **height** |  | cm |  |
|  |  |  |  |
| Left ventricular end-diastolic dimension (LVEDD) |  | mm |  |
| Left ventricular end-systolic dimension (LVESD) |  | mm |  |
| Interventricular septal thickness at end-diastole (IVSd ) |  | mm |  |
| Posterior wall thickness at end-diastole (PWd) |  | mm |  |
| Relative wall thickness (RWT) |  |  | Please specify: |
|  |  |  | 🞏 normal |
|  |  |  | 🞏 concentric hypertrophy |
|  |  |  | 🞏 eccentric hypertrophy |
|  |  |  |  |
| Right ventricular (RV) basal diameter |  | mm | RV enlargement: |
|  |  |  | 🞏 yes |
|  |  |  | 🞏 no |
| Tricuspid annular plane systolic excursion (TAPSE) |  | mm |  |
|  |  |  |  |
| LV end-diastolic volume (A4C) |  | ml |  |
| LV end-systolic volume (A4C) |  | ml |  |
| LV ejection fraction (A4C) |  | % | Visual quantification, if measurement is not possible: |
|  |  |  | 🞏 ≥ 55% |
|  |  |  | 🞏 ≥ 45-54% |
|  |  |  | 🞏 ≥ 30-44% |
|  |  |  | 🞏 < 30% |
|  |  |  |  |
| Left atrial (LA) area |  | cm2 | Left atrial enlargement: |
| Left atrial (LA) volume |  | ml | 🞏 yes |
|  |  |  | 🞏 no |
| Right atrial area |  | cm2 |  |
|  |  |  |  |
| E V_max_ |  | cm/s | Please specify: |
| A V_max_ |  | cm/s | 🞏 normal |
| E/A ratio |  |  | 🞏 impaired filling |
| e´ (septal mitral annular measurement) V_max_ |  | cm/s | 🞏 pseudonormal |
| E/e’ ratio |  |  | 🞏 restrictive |
|  |  |  |  |
| aortic valve mean gradient |  | mmHg |  |
| aortic valve peak gradient |  | mmHg |  |
| aortic valve stenosis |  |  | 🞏 normal |
|  |  |  | 🞏 aortiv valve sclerosis |
|  |  |  | 🞏 mild |
|  |  |  | 🞏 moderate |
|  |  |  | 🞏 severe |
| aortic regurgitation |  |  | 🞏 normal |
|  |  |  | 🞏 mild |
|  |  |  | 🞏 moderate |
|  |  |  | 🞏 severe |
| mitral valve mean gradient |  | mmHg |  |
| mitral stenosis |  |  | 🞏 normal |
|  |  |  | 🞏 mild |
|  |  |  | 🞏 moderate |
|  |  |  | 🞏 severe |
| mitral regurgitation |  |  | 🞏 mild |
|  |  |  | 🞏 moderate |
|  |  |  | 🞏 severe |
|  |  |  | 🞏 mild |
| tricuspid regurgitation (TR) |  |  | 🞏 mild |
|  |  |  | 🞏 moderate |
|  |  |  | 🞏 severe |
|  |  |  | 🞏 mild |
|  |  |  |  |
| peak tricuspid regurgitation (TR) pressure |  | mmHg | If measurement is not possible: |
| systolic pulmonary artery pressure (PAP_sys_) |  | mmHg | 🞏 PAP_sys_ not markedly elevated |
|  |  |  |  |
| resting Left ventricular outflow tract (LVOT) gradient |  | mmHg |  |
| left ventricular outflow tract (LVOT) gradient (Valsalva maneuver) |  | mmHg |  |

**e-Table 3** Participating institutions and principal investigators at each participating institution

| **Participating institution** | **Principal investigator** |
| --- | --- |
| Newcastle upon Tyne Hospitals NHF Foundation Trust, Newcastle upon Tyne, United Kingdom | Dr. Djordje G. Jakovljevic, PhD |
| University Medical Centre Regensburg (Department of Internal Medicine II), Regensburg, Germany | Prof. Dr. Lars S. Maier |
| Institute of cardiovascular diseases of Vojvodina, Sremska Kamenica, Serbia | Prof. Dr. Lazar Velicki |
| Clinical Centre of Serbia (Cardiology Department), Belgrade, Serbia | Prof. Dr. Arsen Ristic |
| Careggi University Hospital, Florence, Italy | Prof. Dr. Iacopo Olivotto |

**References**

1. Borg GA. Psychophysical bases of perceived exertion. Med Sci Sports Exerc 1982;14:377-81.

2. Harding AH, Sargeant LA, Welch A, et al. Fat consumption and HbA(1c) levels: the EPIC-Norfolk study. Diabetes Care 2001;24:1911-6.

3. Ware J, Jr., Kosinski M, Keller SD. A 12-Item Short-Form Health Survey: construction of scales and preliminary tests of reliability and validity. Med Care 1996;34:220-33.

4. Ware JE, Jr., Sherbourne CD. The MOS 36-item short-form health survey (SF-36). I. Conceptual framework and item selection. Med Care 1992;30:473-83.

5. Rector TS, Kubo SH, Cohn JN. Validity of the Minnesota Living with Heart Failure questionnaire as a measure of therapeutic response to enalapril or placebo. Am J Cardiol 1993;71:1106-7.

6. Bilbao A, Escobar A, Garcia-Perez L, Navarro G, Quiros R. The Minnesota living with heart failure questionnaire: comparison of different factor structures. Health Qual Life Outcomes 2016;14:23.

7. Snaith RP, Zigmond AS. The hospital anxiety and depression scale. Br Med J (Clin Res Ed) 1986;292:344.

8. Lang RM, Badano LP, Mor-Avi V, et al. Recommendations for cardiac chamber quantification by echocardiography in adults: an update from the American Society of Echocardiography and the European Association of Cardiovascular Imaging. Eur Heart J Cardiovasc Imaging 2015;16:233-70.
